# Title IX Mandated Reporting: The Views of University Employees and Students

**DOI:** 10.3390/bs8110106

**Published:** 2018-11-20

**Authors:** Amie R. Newins, Emily Bernstein, Roselyn Peterson, Jonathan C. Waldron, Susan W. White

**Affiliations:** 1 Department of Psychology, University of Central Florida, Orlando, FL 32816, USA; Emily.bernstein@knights.ucf.edu (E.B.); rpeterson@knights.ucf.edu (R.P.); 2 Department of Psychology, Virginia Tech, Blacksburg, VA 24061, USA; jwaldron22@vt.edu; 3 Department of Psychology University of Alabama, Tuscaloosa, AL 35487, USA; swwhite1@ua.edu

**Keywords:** Title IX, sexual assault, disclosure, reporting

## Abstract

Per Title IX of the Higher Education Amendments of 1972, many university employees are mandated reporters of sexual assault. University employees (*N* = 174) and students (*N* = 783) completed an online survey assessing knowledge and opinions of this reporting requirement. University employees and students generally reported being quite knowledgeable of reporting requirements. Most university employees indicated they would report an incident disclosed by a student, but students were fairly ambivalent about whether they would disclose to faculty members. Nearly one in five students (17.2%) indicated that Title IX reporting requirements decreased their disclosure likelihood. These findings suggest that mandated reporting policies, as well as how they are presented to students and faculty, should be examined in order to increase compliance and facilitate disclosure.

## 1. Introduction

Sexual assault (i.e., sexual contact that occurs without consent from both parties) is a prevalent issue on university campuses. More than one in five (21 to 23%) college women and more than one in twenty (7 to 11.6%) college men report experiencing attempted or completed sexual assault since beginning college [1,2]. Survivors of sexual assault or rape are at an increased risk of posttraumatic stress disorder, depression, anxiety, and suicidality [3]. The prevalence of sexual assault on college campuses, coupled with the profound negative consequences resulting from such assaults warrants legislative and policy intervention. The current study aims to examine university employee’s and student’s perceptions of one aspect of such a policy: mandated reporting under Title IX.

Title IX of the Higher Education Amendments of 1972 (Title IX) specifies that any educational institution receiving federal funding must prevent sex-based discrimination and respond to acts of sexual discrimination when they do occur [4]. In April 2011, the Office of Civil Rights released a Dear Colleague Letter, which provided specific guidance on schools’ duties to ensure that sexual assault and harassment are properly addressed in educational settings [5]. In particular, this letter explained that universities are obligated to take action in response to sexual violence if any university employees who are not confidential employees (e.g., student health providers, victim services advocates) know of the incident [6]. Specifically, university employees are required to report incidents of sexual violence involving students to the Title IX coordinator. It should be noted that prior to the April 2011 Dear Colleague Letter, Title IX requirements were primarily interpreted through case law [4].

The Clery Act of 1990 requires that universities publish crime statistics. As a result, certain campus authorities are required to track and report anonymous data about crimes on college campuses, but reporting of personally identifying information is not required. In 2013, the Campus Sexual Violence Elimination (SaVE) Act, an amendment to the Clery Act of 1990, was enacted; this law requires universities to report statistics regarding sexual, domestic, and dating violence and to provide training programs to students and employees about these types of violence [7]. The required training programs must include information about Title IX reporting requirements as well as Title IX resources. The Campus SaVE Act also laid out standards for the university’s disciplinary response. Furthermore, some states have elected to go beyond the requirements of Title IX; specifically, some states have implemented laws that require colleges and universities to report sexual assault cases to law enforcement agencies [8]. If other states follow suit, the potential reach of mandatory reporting will expand (i.e., from university employees disclosing to Title IX officials to include further reporting by the Title IX officials to law enforcement), underscoring the importance of understanding knowledge and opinions of reporting requirements. Furthermore, sexual assault is also prevalent among college students internationally [9]; therefore, information about how mandatory reporting laws are perceived could be relevant to other countries considering how to address disclosures of sexual assault and sexual harassment on college and university campuses. For example, Bill 132 in Canada has many similar requirements to Title IX and the Clery Act [10], so the findings of this study may help to inform laws in other countries.

To date, limited research has examined whether university employees (many of whom are mandated reporters under Title IX) and students understand Title IX reporting requirements and whether they agree with these requirements. This line of research is important, as university employees’ perceptions may affect compliance with Title IX, and students’ perceptions likely influence the likelihood that students will disclose to university employees. One previous study examined opinions of victim advocates on college campuses in a state where all campus employees must be mandated reporters, but noted a need for research on the opinions of faculty and other staff [11]. Furthermore, few studies have examined how student characteristics may affect the likelihood of disclosure to a university employee who is required to report incidents under Title IX.

There are several variables that may affect students’ opinions of mandatory reporting. First, rape myth acceptance (RMA; i.e., agreement with inaccurate beliefs about rape) is one factor that may influence students’ Title IX beliefs and their willingness to disclose a sexual assault to university employees. Common rape myths include beliefs that women want to be raped, that men cannot control aggressive sexual impulses, and that many women make false claims about being raped [12]. As a result, individuals high in RMA may be less likely to see a need for mandatory reporting or fear that mandatory reporting could increase the risk of women making false claims about sexual assault experiences. Research in non-collegiate samples has found that individuals high in RMA are less likely to disclose sexual assault experiences to the police [13,14]. Rape myths also tend to involve stereotypes about rape (e.g., that rape is only perpetrated by strangers) [12]; as a result, higher levels of RMA may also affect opinions about mandatory reporting of sexual assault and sexual harassment due to decreased perceived need to address college sexual assaults, which often do not fit stereotypes (e.g., the majority of sexual assaults are perpetrated by known others) [15]. Furthermore, feminist theory dictates that RMA allows sexual assault to continue to occur in American culture [16]; therefore, feminist beliefs may be another variable that affects students’ disclosure likelihood, as individuals high in feminist beliefs may be more likely to reject rape myths. In fact, a recent study found that universities with a female president reported 40% more rapes to the Department of Education and had higher levels of estimated compliance with reporting than universities with a male president [17].

In addition, prior research has shown that men who have perpetrated sexual assault report higher levels of hostility toward women and more acceptance of using verbal pressure in sexual encounters compared to men who have not perpetrated sexual assault [18]. Individuals who engage in aggressive behaviors may be more accepting of sexual aggression, which could affect acceptance of mandatory reporting requirements. Therefore, it is possible that aggression would also relate to Title IX perceptions among students.

Finally, previous research has shown that sexual assault history influences likelihood of disclosure [19]. Individuals with a history of sexual assault have had to work through decisions about to whom they disclose. Furthermore, they likely experienced a range of social reactions to their disclosures [20,21,22], which likely color their opinions on how disclosure information should be handled. 

To the authors’ knowledge, only one previous study has examined knowledge and opinions of Title IX reporting among university employees and students [23]. However, the data collection for that study occurred in 2013 (i.e., prior to implementation of the Campus SaVE Act). Newins and White (2018) found that both university employees and students generally reported high levels of knowledge and agreement with Title IX reporting requirements. When provided with a vignette describing a student’s disclosure of sexual assault perpetrated by another student, most employees (78.9%) indicated they would likely report the incident to the Title IX coordinator. A substantial proportion of students indicated they would tell a faculty member if they knew of a sexual assault perpetrated by another student (47.1%) and if they experienced a sexual assault (40.2%). Among students, RMA was negatively associated with agreement with knowledge and opinion questions about Title IX reporting requirements. While feminist beliefs were not related to those ratings, they were positively associated with the estimated likelihood of disclosing one’s own sexual assault to a university employee. Finally, compared to students who did not report a history of sexual assault, students who were survivors of sexual assault were more likely to believe that reporting sexual assault perpetrated by a student should be required, less likely to believe that reporting of sexual harassment perpetrated by a student should be required, more likely to indicate they would disclose sexual assault involving third parties, more likely to be unsure whether they would report their own sexual assault, and more likely to say that Title IX reporting requirements decreased the likelihood that they would tell a university employee about their own sexual assault.

The current paper sought to extend these findings by examining whether there are differences in knowledge and opinions of Title IX reporting requirements following implementation of the Campus SaVE Act. Additionally, this study sought to replicate and extend the findings regarding predictors of Title IX opinions among students by adding aggression. Research on employee and student knowledge and opinions of mandated reporting of sexual assault is needed to inform policy. In particular, the assumptions that underlie mandated reporting have limited empirical support [24]; therefore, further research on perceptions of mandated reporting is needed to inform the future of these policies. We expected that the current study would provide additional evidence that university employees and students know about reporting requirements. The following hypotheses were proposed.

**Hypothesis** **1**: Given that the Campus SaVE Act requires additional education about sexual assault on college campuses, university employees and students in the current study were expected to be more aware of reporting requirements and be more likely to agree with mandated reporting compared to employees and students who completed a similar study prior to implementation of the Campus SaVE Act.

**Hypothesis** **2**: Due to the power differential between faculty and students, we expected more agreement with mandated reporting when sexual harassment and sexual assault is perpetrated by a faculty member. We also expected more agreement with mandated reporting for cases of sexual assault compared to sexual harassment, as sexual assault is typically perceived as a more severe form of victimization.

**Hypothesis** **3**: Given the literature cited above, among students, RMA and aggression were expected to be negatively associated with agreement with mandated reporting, and feminist beliefs were expected to be positively associated with agreement with mandated reporting.

**Hypothesis** **4**: Based on previous research, among students, we hypothesized that survivors of sexual assault would be less likely to indicate they would disclose sexual assault compared to students without a history of sexual assault.

## 2. Materials and Methods

### 2.1. Participants and Procedures

The study was approved by the university Institutional Review Board. University employees were recruited through recruitment emails sent to academic department heads and via listservs. Students were recruited through the Psychology Department research management website. It should be noted that all undergraduate students enrolled in psychology classes are eligible to participate in the research management system, and many non-psychology majors enroll in psychology courses for general education requirements, for courses required by other majors, and to fulfill elective requirements. Data collection occurred during the Spring 2015 semester. During the data collection period, all University employees had mandatory Title IX training, which could be completed either in person or online. This training informed employees that all employees (with few exceptions, e.g., counseling center staff) were mandated reporters. As such, all employees should have been familiar, prior to data collection, with the Title IX reporting mandate. University employees were not compensated for participating; students received extra credit in psychology courses. Participants implied consent to participate by entering the survey after being provided with information about risks and benefits. As part of the consent document, participants were informed that data collection was anonymous. All data were collected via anonymous online surveys hosted on SurveyMonkey^®^.

Participants were 174 university employees and 783 college students at a large public university who completed the online survey. University employees had a mean age of 42.92 (*SD* = 13.72; range: 21 to 86), completed their highest degree an average of 14.31 years (*SD* = 12.82) prior to completing the survey, and had been working in a university setting for an average of 11.68 years (*SD* = 11.40). The majority of the sample identified their gender as female (71.8%) and race as White (89.1%); four university employees (2.3%) identified their ethnicity as Hispanic or Latino(a). University employees held a range of positions (28.2% professors of various ranks (assistant, associate, and full), 22.4% administrators, 18.4% graduate teaching assistants, and 31.0% other positions (e.g., adjunct professors, instructors)).

Students had a mean age of 19.83 (*SD* = 1.56; range: 17 to 41). Most student participants identified as female (77.8%) and White (82.8%). Participants were primarily in their first year (35.8%), followed by second year (24.9%), third year (22.0%), fourth year (15.8%), and fifth year or beyond (1.5%). Two- hundred-eighty students (35.8%) indicated experiencing at least one sexual assault (attempted or completed) since the age of 14.

### 2.2. Measures

Both university employees and students completed the Title IX Knowledge and Opinions Questionnaire; only students completed the remaining measures due to a desire to keep the university employee survey as brief as possible to increase completion.

Title IX Knowledge and Opinions Questionnaire. University employees and students were asked about knowledge and opinions of university employees’ reporting responsibilities related to four student disclosure scenarios: (1) sexual harassment by a student, (2) sexual harassment by a faculty/staff member, (3) rape by a student, and (4) rape by a faculty/staff member. This questionnaire was identical to the one used by Newins and White [23]. Employees were asked about their reporting requirements if a student were to disclose each scenario, and students were asked about reporting requirements if they were to disclose that they had experienced each scenario. Participants were asked to rate agreement with three statements using a seven-point Likert scale (1 = strongly disagree to 7 = strongly agree): (1) university employees are required to report the incident to university officials (i.e., knowledge of reporting requirement); (2) university employees should be required to report the incident to university officials, even if the student states they do not want the incident reported after disclosing it (i.e., opinion of non-consented reporting); and (3) university employees should be required to facilitate reporting if the student wishes to report it to university officials (i.e., opinion of consented reporting).

A brief description of Title IX mandated reporting was then provided. Then, university employees were asked to indicate how likely they would be to report a student disclosure of sexual assault to the Title IX coordinator. Students were asked to rate how likely they would be to (1) tell a faculty member if they had been sexually assaulted by another student, and (2) tell a faculty member if they knew an acquaintance had perpetrated a sexual assault against another student. The rating scale ranged from 1 (definitely would not) to 5 (definitely would). Students were then asked to rate the degree to which the likelihood of disclosing their own sexual assault to a university employee was affected by knowing the university employee reporting requirements on a scale from 1 (much less likely to tell) to 5 (much more likely to tell).

Feminist Perspectives Scale (FPS) [25]: The FPS consists of 50 items assessing “liberal,” “radical,” “social,” “cultural,” and “women of color” feminism. Participants were asked to rate how much they agreed with the statements from 1 (strongly disagree) to 7 (strongly agree). Scores are summed, with higher total scores indicating stronger feminist perspectives. Previous research demonstrated good internal consistency (α = 0.92) and good convergent and divergent validity in a college sample [12]. For the current study, Cronbach’s alpha was 0.94.

Illinois Rape Myth Acceptance Scale (IRMAS) [12]: On the IRMAS, students were asked to rate how much they agreed with each of the 45 rape myths on a 1 (not at all agree) to 7 (very much agree) scale. Scores are summed, with higher total scores indicating higher myth acceptance. Previous research demonstrated good internal consistency (α = 0.93) and construct validity [12]. For the current study, Cronbach’s alpha was 0.93.

Sexual Experiences Survey—Short Form Victimization (SES-SFV) [26]: On the SES-SFV, women reported on seven types of unwanted sexual experiences (i.e., sexual touching/contact, oral sex, vaginal sex, anal sex, attempted oral sex, attempted vaginal sex, and attempted anal sex), and men were asked about five types of unwanted sexual experiences (excluding questions related to vaginal sex). Participants were asked to indicate whether or not they had experienced each incident as a result of five different coercion strategies during two non-overlapping periods: (1) during the 12 months prior to completing the survey, and (2) during the period between their 14th birthday and 12 months prior to completing the survey. Test–retest reliability, convergent validity, and concurrent validity of data from the SES-SFV have been demonstrated [27]. For the current study, participants were classified as either having experienced sexual victimization since the age of 14 (i.e., endorsed any item on the SES-SFV) or not (i.e., denied all items on the SES-SFV).

Aggression Questionnaire (AQ) [28]: The AQ consists of 29 items that measure aggressive tendencies. Participants were asked to rate how characteristic each item is of them from 1 (extremely uncharacteristic of me) to 5 (extremely characteristic of me). The AQ consists of four subscales: physical aggression, verbal aggression, anger, and hostility. Subscale scores are created by summing the items for each subscale. Previous research has demonstrated that the internal consistency of the four subscales and total score ranged from 0.72 to 0.89 [28]. For the current study, Cronbach’s alphas ranged from 0.77 to 0.83.

Impulsive/Premeditated Aggression Scale (IPAS) [29]: The IPAS is a 30-item measure of impulsive and premeditated aggression. Participants are asked to rate their agreement with each statement based on the last 6 months from 1 (strongly disagree) to 5 (strongly agree). Subscale scores (i.e., impulsive aggression and premeditated aggression) are computed, and higher scores indicate higher aggression. In a college sample, the IPAS demonstrated high internal consistency (impulsive aggression α = 0.77; premeditated aggression α = 0.81) [30]. For the current study, Cronbach’s alphas were 0.72 for impulsive aggression and 0.87 for premeditated aggression.

### 2.3. Data Analyses

All data were entered into Statistical Package for Social Sciences (SPSS) Version 23 (IBM Corp. Released 2015. IBM SPSS Statistics for Windows, Version 23.0. Armonk, NY: IBM Corp.) for analysis. Independent samples Mann–Whitney U-tests were used to compare responses from the current study with those from a study that used the same Title IX Knowledge and Opinions Questionnaire and was conducted prior to implementation of the Campus SaVE Act at the same university [23]. Related-samples Wilcoxon signed rank tests were used to examine whether knowledge and opinions of reporting requirements differed by perpetrator category (student vs faculty), type of sexual victimization (sexual harassment vs rape), and student consent for reporting (non-consented vs consented). Spearman’s rho correlations were used to examine the relationship between student responses on the Title IX Knowledge and Opinions Questionnaire and measures of feminist beliefs, RMA, and aggression. In order to examine whether survivors of sexual assault differed in their knowledge and opinions regarding Title IX mandated reporting as well as their estimated likelihood of disclosure, responses to these items were trichotomized, and multinomial logistic regressions were run to examine sexual assault history as a predictor of responses to these items (participant gender was entered as a covariate to control for gender differences).

## 3. Results

### 3.1. University Employees

#### 3.1.1. Descriptive Information

Means, standard deviations, and medians for responses to each of the twelve knowledge and opinion items are presented in Table 1. University employees generally reported high levels of knowledge of reporting requirements in instances of sexual harassment by another student, sexual harassment by a faculty member, rape by another student, and rape by a faculty member. University employees were generally in agreement with both non-consented and consented reporting in instances of sexual harassment by another student, sexual harassment by a faculty member, rape by another student, and rape by a faculty member. Additionally, most university employees (84.5%) indicated that they would report a student disclosure of sexual assault by another student to the Title IX coordinator (*M* = 4.44, *SD* = 0.93, Median = 5.00). 

#### 3.1.2. Comparisons with Previous Research

Hypothesis 1 was partially supported. University employees reported stronger agreement with the statement assessing knowledge of reporting requirements in instances of sexual harassment by a faculty member in the current study compared to the previous study (Newins & White, 2018 [23]; Prior study: *M* = 6.25, *SD* = 1.46, Median = 7.00; *z* = 2.12, *p* = 0.034, *η2* = 0.02). There were no significant differences between the two studies on ratings for the other eleven items assessing knowledge and opinions of mandated Title IX reporting (*z*s = −1.39 to 1.89, *p*s > 0.059; see Newins & White, 2018 [23], for descriptive statistics from the prior study). University employees were also more likely to indicate that they would report a student disclosure of sexual assault in the current study (Prior study: *M* = 4.21, *SD* = 1.08, Median = 4.00; *z* = 2.07, *p* = 0.038, *η2* = 0.01).

#### 3.1.3. Comparisons by Perpetrator Category, Type of Sexual Victimization, and Student Consent for Reporting

Hypothesis 2 was mostly supported. University employees indicated greater knowledge of the requirement to report and higher agreement with required reporting regardless of student wishes when the hypothetical incident was perpetrated by faculty members compared to those perpetrated by students (*z*s = 2.67 to 6.37, *p*s < 0.009, *r*s = 0.14 to 0.34). They indicated more agreement with being required to assist students with reporting sexual harassment perpetrated by faculty members compared to incidents perpetrated by students (*z* = 2.55, *p* < 0.001, *r* = 0.14), but there was no difference by perpetrator type in agreement with being required to assist students with reporting incidents of rape (*z* = 1.49, *p* = 0.136, *r* = 0.08).

University employees indicated greater knowledge of mandated reporting and stronger agreement with required reporting regardless of student wishes when the hypothetical incident involved rape, compared to sexual harassment (*z*s = 2.33 to 5.76, *p*s < 0.021, *r*s = 0.12 to 0.31). They indicated more agreement with being expected to assist with reporting incidents of rape (compared to incidents of sexual harassment) when the hypothetical perpetrator was a student (*z* = 3.50, *p* < 0.001, *r* = 0.19), but there was no difference in responses to this item by incident type when the perpetrator was a faculty member (*z* = 1.38, *p* = 0.168, *r* = 0.07).

University employees indicated greater agreement with non-consented reporting (vs consented reporting) for incidents of rape when a faculty member was the perpetrator (*z* = −2.32, *p* =0.021, *r* = 0.12). Opinions of consented and non-consented reporting did not differ for the other three situations (*z*s = −1.21 to 1.39, *p*s > 0.163).

### 3.2. Students

#### 3.2.1. Descriptive Information

Descriptive statistics for the twelve Title IX knowledge and opinion questions are presented in Table 1. Similar to university employees, students generally reported high levels of knowledge of, and agreement with, mandated reporting of sexual violence involving student survivors. Students varied in their indicated likelihood of disclosing their own hypothetical sexual assault to faculty members (19.9% indicated they would not disclose, 35.5% were unsure, and 44.6% indicated they would disclose; *M* = 3.41, *SD* = 1.14, Median = 3.00) and a hypothetical sexual assault involving other students (14.7% indicated they would not disclose, 37.0% were unsure, and 48.3% indicated they would disclose; *M* = 3.47, *SD* = 1.09, Median = 3.00). Similarly, students varied in how mandated reporting requirements influenced their disclosure likelihood (17.2% indicated they were less likely to disclose, 53.9% indicated it was unchanged, and 28.9% indicated they were more likely to disclose; *M* = 3.18, *SD* = 1.00, Median = 3.00).

#### 3.2.2. Comparisons with Previous Research 

Hypothesis 1 was partially supported; however, some results were contrary to Hypothesis 1. Students indicated stronger agreement with the statement assessing their opinion of faculty members being required to assist students with reporting incidents of sexual harassment perpetrated by another student in the current study compared to the previous study (Newins & White, 2018 [23]; Prior study: *M* = 5.89, *SD* = 1.51, Median = 7.00; *z* = 2.53, *p* = 0.012, *η2* = 0.003). However, students indicated less agreement with mandated reporting of sexual harassment perpetrated by faculty members and rape perpetrated by both students and faculty members (Prior study: *M* = 5.84, *SD* = 1.59, Median = 6.00, *M* = 5.68, *SD* = 1.71, Median = 6.00, *M* = 6.10, *SD* = 1.56, Median = 7.00, respectively; *z*s = −3.84 to −2.16, *p*s < 0.032, *η2*s = 0.003 to 0.009). There were no significant differences between the two studies on ratings for the other eight items assessing knowledge and opinions of mandated Title IX reporting or on the items assessing estimated disclosure likelihood (*z*s = −1.72 to 1.71, *p*s > 0.084; see [23] for descriptive statistics from prior study).

#### 3.2.3. Comparisons by Perpetrator Category, Type of Sexual Victimization, and Student Consent for Reporting

Hypothesis 2 was supported. Student ratings for all three items were higher for incidents of sexual harassment and rape when the hypothetical perpetrator was a faculty member rather than a student (*z*s = 4.71 to 12.99, *p*s < 0.001, *r*s = 0.12 to 0.33). Students indicated more agreement with both opinion statements (i.e., mandated reporting and consented reporting) for incidents of rape compared to sexual harassment (*z*s = 4.54 to 10.98, *p*s < 0.001, *r*s = 0.11 to 0.28). Students were more likely to indicate awareness of mandated reporting for rape (vs sexual harassment) when the perpetrator was a student (*z* = 6.77, *p* < 0.001, *r* = 0.17), but responses about awareness of mandated reporting did not differ significantly between rape and sexual harassment when the perpetrator was a faculty member (*z* = 1.85, *p* = 0.064, *r* = 0.05). Students indicated greater agreement with consented reporting (vs non-consented reporting) in all four situations (*z*s = 9.33 to 14.15, *p*s < 0.001, *r*s = 0.24 to 0.36).

#### 3.2.4. Relationships Between Title IX Reporting Questions and Participant Characteristics

Correlations are presented in Table 2. Hypothesis 3 was mostly supported. Feminist beliefs were positively correlated with knowledge of mandated reporting for three of the scenarios and with beliefs that faculty members should be required to assist students with reporting when the student wants to report in two scenarios. RMA was negatively associated with almost all of the Title IX knowledge and opinion questions, and it was negatively associated with likelihood of disclosing a third-party sexual assault to faculty members. While there was some variability, scores from the various aggression scales, particularly physical aggression and hostility, were generally negatively associated with ratings on the Title IX knowledge and opinion questions; however, correlations were small. 

#### 3.2.5. Differences in Ratings on Title IX Reporting Questions by History of Sexual Assault.

Hypothesis 4 was supported. Frequencies for participant response categories are presented in Table 3; results of the multinomial logistic regressions are presented in Table 4. Sexual assault was not associated with responses to any of the Title IX knowledge or opinion questions. However, sexual assault survivors were more than twice as likely as students without a history of sexual assault to indicate they would not disclose (vs would disclose) their own sexual assault to a faculty member; the odds of being unsure (vs would disclose) did not differ based on sexual assault history. Additionally, compared to students who did not report a history of sexual assault, survivors of sexual assault were more than two times more likely to indicate that knowledge of mandated reporting decreased their likelihood of disclosure (vs increased); the odds of no change (vs increased likelihood of disclosure) did not differ based on sexual assault history. Finally, individuals who experienced sexual assault were nearly one and a half times more likely to be unsure (vs would disclose) if they would disclose a sexual assault involving third parties; odds of indicating they would not disclose (vs would disclose) did not differ.

## 4. Discussion

Results indicated that university employees and students are aware of mandated reporting of sexual violence and generally agree with mandated reporting in theory. The vast majority (84.5%) of sampled university employees indicated they would report a student disclosure of sexual assault (after being informed of mandated reporting requirements); however, 10.3% of university employees were unsure if they would report and 5.2% indicated they would not report. A substantial proportion of students indicated ambivalence about whether they would disclose a sexual assault (both if they were the survivor and if it involved student third parties) to a faculty member with over one-third being unsure if they would report in both scenarios and nearly one-fifth indicating they would not disclose their own sexual assault. While just over half of students (53.9%) indicated that mandated reporting requirements did not affect the likelihood that they would disclose to a faculty member if they experienced a sexual assault, a substantial minority (17.2%) indicated these requirements decreased their disclosure likelihood.

The results of the current study reveal that some university employees are hesitant to report despite knowledge of the requirement, a meaningful proportion of students say that mandated reporting decreases their disclosure likelihood, and students who have experienced a sexual assault are less likely to indicate they would disclose their sexual assault to a mandated reporter and more likely to indicate that mandated reporting disclosure decreases their disclosure likelihood. Taken together, this information suggests that more research on this topic is needed in order to inform future policies related to sexual assault reporting. In particular, research is needed to determine whether mandated reporting of sexual assault at the university level is associated with benefits to survivors and the broader university community. Furthermore, research is needed to determine if policies should be modified (see Holland et al., 2018, for discussion of alternative policy options) [24].

When the results of the current study were compared with a prior study on the topic [23], there were a few important differences in university employees’ and students’ opinions regarding Title IX. It should be noted that although the two studies were conducted at the same university, the prior study was conducted prior to required compliance with the Campus SaVE Act. At the time of the prior study, Title IX training was optional for university employees; however, it was mandatory at the time of the current study. It is encouraging that university employees in the current study were more likely to indicate they would report to the Title IX coordinator if a student disclosed sexual assault to them, but the effect size was very small. Students in the current sample had lower levels of agreement with mandated reporting in three of the four hypothetical scenarios (i.e., sexual harassment by a faculty member and rape by a student or faculty member). While the effect sizes were small, these results suggest that caution should be taken with policies requiring mandated reporting on college campuses. If agreement with mandated reporting is declining, student disclosures could decrease. Therefore, further examination of the effects of mandated reporting on disclosure and compliance with investigations is warranted.

### 4.1. Correlates of Title IX Opinions among Students

RMA was negatively associated with responses on most of the Title IX questions. This finding is not surprising, since RMA involves trivializing the experience of rape, excusing the perpetrator, and blaming the survivor [12,16]. Furthermore, it is consistent with research that has revealed that acceptance of certain rape myths is associated with unacknowledged rape among college women; specifically, women who experienced a rape but did not label it as such (i.e., were unacknowledged) endorsed higher levels of RMA for myths involving rape stereotypes and myths that men do not intend to rape [31]. Given that RMA affects how survivors label their experiences, it is not surprising that it also affects whether college students believe sexual assault and sexual harassment warrant mandated reporting. In contrast to the previous study, feminist beliefs were not related to items assessing disclosure likelihood, but they were positively associated with some of the Title IX knowledge and opinion questions, particularly the knowledge question. Given that Title IX prohibits discrimination on the basis of sex and gender [4], it is not surprising that individuals who endorse higher levels of feminist beliefs would be more aware of what this act requires. Aggression was generally negatively associated with knowledge and opinions of mandated reporting, with the most consistent findings being for physical aggression and hostility. Prior research has shown that hostility and acceptance of aggression in sexual encounters are positively associated with the likelihood of sexual assault perpetration among men [18], so it is unsurprising that aggression would be negatively associated with opinions of mandated reporting for sexual violence. It is possible that individuals who engage in more frequent aggressive behaviors are more likely to either have perpetrated or know someone who has perpetrated a sexual assault, which could influence beliefs about how disclosures of sexual assault should be handled.

Survivors of sexual assault were more likely to indicate they would not disclose to a faculty member if they were sexually assaulted and were more likely to be unsure if they would disclose knowledge of a sexual assault involving third party students. In addition, survivors of sexual assault were more likely to indicate that mandated reporting requirements decreased their disclosure likelihood. These findings are generally consistent with results of the previous study [23]. Additionally, in another study, college women who experienced an adolescent sexual assault were less likely to disclose a later sexual assault than their peers who did not experience a prior sexual assault [19]. These findings underscore the importance of working to ensure that survivors have a positive disclosure experience in order to increase the likelihood of later disclosure. 

### 4.2. Practical Implications

A recent review of the assumptions underlying mandated reporting found relatively limited evidence to support these assumptions [24]. Specifically, they identified both supporting and contradictory evidence that mandated reporting increases knowledge of the prevalence of sexual assault and that mandatory reporting benefits survivors, employees, and institutions. Furthermore, several publications underscore concerns that mandated reporting on college campuses may result in unintended negative consequences including loss of control of disclosure [32], possible decreases in disclosures [11], secondary victimization [33], and possible victim-blaming during the adjudication process [32]. Furthermore, mandated reporting raises ethical issues for psychologists and others in academic settings [23,24]. The current findings suggest that although the majority of students and university employees are aware of mandated reporting under Title IX, there is variability in agreement with these mandates. It is possible that addressing rape myths and fostering feminist beliefs may increase agreement with these mandates. However, given that individuals who endorsed a history of sexual assault were less likely to say they would disclose to a faculty member, it is important that Title IX officials talk with survivors to identify ways of improving the Title IX investigation process and to identify ways to increase disclosure.

### 4.3. Limitations

First, data were collected at a single university, thereby limiting ability to generalize to other schools; however, this limitation did allow for a cleaner comparison to the prior study, which was conducted at the same institution [23]. Additionally, the use of predominately White convenience samples limits the generalizability of the results. Furthermore, for comparisons pre- and post-implementation of the Campus SaVE Act, it would have been ideal to have followed a single sample longitudinally. In addition, an a priori power analysis was not conducted to determine the necessary sample size. However, post-hoc power analyses revealed that the obtained sample sizes provided power of greater than 0.80 to detect a medium effect in the multinomial logistic regressions and power of at least 0.90 to detect medium effect sizes in all other analyses. An additional limitation is the fact that correlates of Title IX knowledge and opinions could not be examined for university employees, as additional measures were not included in their version of the survey. Finally, data on prior familiarity with the Title IX requirements and completion of trainings related to mandated reporting was not collected; however, given that training was required when these data were collected, we assume most, if not all, participants had received formal training.

### 4.4. Future Directions

Given the recent increase in attention to sexual assault both in the news media and on social media (e.g., the *#MeToo* movement), more recent research examining disclosure are needed. Furthermore, because this attention may influence disclosures that may involve mandated reporting, future research will be needed to determine whether perceptions of mandated reporting are affected. Furthermore, these findings should be replicated in more diverse samples to ensure generalizability to a wider range of college students. Finally, international research on the acceptability of mandated reported is needed, particularly in countries considering implementing mandated reporting laws.

## 5. Conclusions

The spirit and intention of the Title IX reporting requirements are broadly considered a step toward the goal of decreasing incidents of sexual aggression and harassment on college campuses. These results suggest, however, that general campus-wide training initiatives, which include education on the Title IX requirements, do not necessarily increase the likelihood of complying with mandated reporting. A sizeable minority of students even indicate that knowledge that university employees are mandated reporters decreases their likelihood of disclosing an incident. Also, consistent with prior research, within-person variables, such as RMA, hostility, and sexual assault history, predict disclosure likelihood. Collectively, these results indicate that awareness-building and training initiatives may have more sustained and positive impact when some of the reasons for non-disclosure are considered and addressed directly.

## Figures and Tables

**Table 1:** Descriptive statistics for Title IX knowledge and opinion questions.

		University Employees *N* = 174			Students *N* = 783		
Scenario	Statement	*M*	*SD*	Median	*M*	*SD*	Median
Student was sexually harassed by another student	Knowledge of Reporting Requirement	5.99	1.79	7.00	5.82	1.56	6.00
	Opinion of Non-Consented Reporting	4.90	2.09	5.50	4.93	1.82	5.00
	Opinion of Consented Reporting	5.20	2.12	6.00	6.07	1.43	7.00
Student was sexually harassed by a faculty member	Knowledge of Reporting Requirement	6.36	1.64	7.00	6.27	1.32	7.00
	Opinion of Non-Consented Reporting	5.64	2.02	7.00	5.67	1.67	6.00
	Opinion of Consented Reporting	5.42	2.11	7.00	6.25	1.34	7.00
Student was raped by another student	Knowledge of Reporting Requirement	6.33	1.67	7.00	6.05	1.50	7.00
	Opinion of Non-Consented Reporting	5.47	2.13	7.00	5.45	1.76	6.00
	Opinion of Consented Reporting	5.44	2.16	7.00	6.25	1.35	7.00
Student was raped by a faculty member	Knowledge of Reporting Requirement	6.47	1.56	7.00	6.32	1.30	7.00
	Opinion of Non-Consented Reporting	5.89	1.99	7.00	5.86	1.63	7.00
	Opinion of Consented Reporting	5.50	2.15	7.00	6.37	1.29	7.00

Note: Ratings for all questions ranged from 1 (*strongly disagree*) to 7 (*strongly agree*). Knowledge of Reporting Requirement = I am (Faculty/staff members are) required to report the incident to university officials; Opinion of Non-Consented Reporting = I (Faculty/staff members) should be required to report the incident to university officials, even if the student does (I do) not want the incident reported; Opinion of Consented Reporting = I (Faculty/staff members) should be required to help the student report the incident to university officials, if the student wants (I want) to do so.

**Table 2:** Correlations of Title IX questions and reporting likelihood questions with FPS and IRMAS scores (student sample; *N* = 783).

Question	FPS	IRMAS	AQ-PA	AQ-VA	AQ-Anger	AQ-Hostility	IPAS-Impulsive	IPAS-Premed.
I disclosed being sexually harassed by another student								
Knowledge of Reporting Requirement	0.07	−0.09 *	−0.10 **	−0.08 *	−0.08 *	−0.07 *	−0.04	0.02
Opinion of Non-Consented Reporting	0.03	−0.02	−0.11 **	−0.08 *	−0.05	−0.08 *	−0.05	−0.06
Opinion of Consented Reporting	0.08 *	−0.13 ***	−0.15 **	−0.02	−0.06	−0.08 *	−0.06	−0.04
I disclosed being sexually harassed by a faculty member								
Knowledge of Reporting Requirement	0.10 *	−0.15 ***	−0.15 ***	−0.06 *	−0.07	−0.09 *	−0.08 *	−0.00
Opinion of Non-Consented Reporting	0.03	−0.11 **	−0.10 **	−0.06	−0.07	−0.09 *	−0.06	−0.08 *
Opinion of Consented Reporting	0.08	−0.15 ***	−0.15 ***	−0.01	−0.10 **	−0.11 **	−0.08 *	−0.01
I disclosed being raped by another student								
Knowledge of Reporting Requirement	0.07 *	−0.08 *	−0.11 **	−0.08 *	−0.13 ***	−0.10 **	−0.04	0.02
Opinion of Non-Consented Reporting	−0.01	−0.06	−0.09 **	−0.08 *	−0.06	−0.09 *	−0.08 *	−0.09 *
Opinion of Consented Reporting	0.07	−0.16 ***	−0.12 **	−0.02	−0.08 *	−0.09 *	−0.06	−0.02
I disclosed being raped by a faculty member								
Knowledge of Reporting Requirement	0.07 *	−0.10 **	−0.11 **	−0.06	−0.11 **	−0.06	−0.05	0.02
Opinion of Non-Consented Reporting	−0.03	−0.07 *	−0.07 *	−0.05	−0.09 *	−0.08 *	−0.08 *	−0.07
Opinion of Consented Reporting	0.07 *	−0.14 ***	−0.12 **	0.02	−0.07	−0.07	−0.08 *	−0.01
If you were sexually assaulted by another student, how likely would you be to tell a faculty member about the incident?	0.01	−0.05	−0.03	0.01	−0.03	−0.13 ***	−0.08 *	−0.10 **
If you knew that an acquaintance of yours had sexually assaulted another student, how likely would you be to tell a faculty member about the incident?	0.01	−0.11 **	−0.01	0.03	−0.03	−0.03	−0.07*	−0.11 **
Is the likelihood of you telling a trusted faculty member if you were sexually assaulted affect by Title IX reporting requirements?	0.01	0.00	−0.05	−0.08 *	−0.07 *	−0.12 **	0.00	−0.07 *

Note: FPS = Feminist Perspectives Scale; IRMAS = Illinois Rape Myth Acceptance Scale; AQ = Aggression Questionnaire; PA = Physical Aggression; VA = Verbal Aggression; IPAS = Impulsive-Premeditated Aggression Scales; Premed. = Premeditated; Knowledge of Reporting Requirement = Faculty/staff members are required to report the incident to university officials; Opinion of Non-Consented Reporting = Faculty/staff should be required to report the incident to university officials, even if I do not want the incident reported; Opinion of Consented Reporting = Faculty/staff should be required to help me report the incident to university officials, if I want to do so. * *p* < 0.05, ** *p* < 0.01, *** *p* < 0.001.

**Table 3:** Student responses by sexual assault history (*N* = 783).

Question	No History of Sexual Assault *N* = 503		History of Sexual Assault *N* = 280	
	*n*	%	*n*	%
I disclosed being sexually harassed by another student				
Knowledge of Reporting Requirement				
Disagree (ratings of 1 to 3)	37	7.4	32	11.4
Neutral (rating of 4)	59	11.7	23	8.2
Agree (ratings 5 to 7)	407	80.9	225	80.4
Opinion of Non-Consented Reporting				
Disagree (ratings of 1 to 3)	102	20.3	73	26.1
Neutral (rating of 4)	79	15.7	44	15.7
Agree (ratings 5 to 7)	322	64.0	163	58.2
Opinion of Consented Reporting				
Disagree (ratings of 1 to 3)	39	7.8	16	5.7
Neutral (rating of 4)	27	5.4	18	6.4
Agree (ratings 5 to 7)	437	86.9	246	87.9
I disclosed being sexually harassed by a faculty member				
Knowledge of Reporting Requirement				
Disagree (ratings of 1 to 3)	24	4.8	12	4.3
Neutral (rating of 4)	30	6.0	14	5.0
Agree (ratings 5 to 7)	449	89.3	254	90.7
Opinion of Non-Consented Reporting				
Disagree (ratings of 1 to 3)	61	12.1	32	11.4
Neutral (rating of 4)	50	9.9	25	8.9
Agree (ratings 5 to 7)	392	77.9	223	79.6
Opinion of Consented Reporting				
Disagree (ratings of 1 to 3)	29	5.8	15	5.4
Neutral (rating of 4)	20	4.0	9	3.2
Agree (ratings 5 to 7)	454	90.3	256	91.4
I disclosed being raped by another student				
Knowledge of Reporting Requirement				
Disagree (ratings of 1 to 3)	33	6.6	25	8.9
Neutral (rating of 4)	36	7.2	20	7.1
Agree (ratings 5 to 7)	434	86.3	235	83.9
Opinion of Non-Consented Reporting				
Disagree (ratings of 1 to 3)	76	15.1	50	17.9
Neutral (rating of 4)	52	10.3	26	9.3
Agree (ratings 5 to 7)	375	74.6	204	72.9
Opinion of Consented Reporting				
Disagree (ratings of 1 to 3)	25	5.0	16	5.7
Neutral (rating of 4)	28	5.6	11	3.9
Agree (ratings 5 to 7)	450	89.5	253	90.4
I disclosed being raped by a faculty member				
Knowledge of Reporting Requirement				
Disagree (ratings of 1 to 3)	25	5.0	14	5.0
Neutral (rating of 4)	24	4.8	11	3.9
Agree (ratings 5 to 7)	454	90.3	255	91.1
Opinion of Non-Consented Reporting				
Disagree (ratings of 1 to 3)	50	9.9	26	9.3
Neutral (rating of 4)	51	10.1	20	7.1
Agree (ratings 5 to 7)	402	79.9	234	83.6
Opinion of Consented Reporting				
Disagree (ratings of 1 to 3)	27	5.4	13	4.6
Neutral (rating of 4)	18	3.6	6	2.1
Agree (ratings 5 to 7)	458	91.1	261	93.2
If you were sexually assaulted by another student, how likely would you be to tell a faculty member about the incident?				
Would not disclose (ratings of 1 or 2)	82	16.3	74	26.4
Unsure (rating of 3)	176	35.0	102	36.4
Would disclose (ratings of 4 or 5)	245	48.7	104	37.1
If you knew that an acquaintance of yours had sexually assaulted another student, how likely would you be to tell a faculty member about the incident?				
Would not disclose (ratings of 1 or 2)	66	13.1	49	17.5
Unsure (rating of 3)	177	35.2	113	40.4
Would disclose (ratings of 4 or 5)	260	51.7	118	42.1
Is the likelihood of you telling a trusted faculty member if you were sexually assaulted affect by Title IX reporting requirements?				
Decreased likelihood (ratings of 1 or 2)	70	13.9	65	23.2
Unchanged (rating 3)	274	54.9	146	52.1
Increase likelihood (ratings of 4 or 5)	157	31.2	69	24.6

Note: Knowledge of Reporting Requirement = Faculty/staff members are required to report the incident to university officials; Opinion of Non-Consented Reporting = Faculty/staff should be required to report the incident to university officials, even if I do not want the incident reported; Opinion of Consented Reporting = Faculty/staff should be required to help me report the incident to university officials, if I want to do so.

**Table 4:** Results of multinomial logistic regressions examining the effect of sexual assault history on responses to Title IX questions (student sample; *N* = 783).

Question and Response	*Nagelkerke Psuedo-R^2^*	*b*	*SE*	*Wald χ^2^(1)*	*p*	*OR*	95% *CI* for *OR*
I disclosed being sexually harassed by another student							
Knowledge of Reporting Requirement	0.01						
Disagree (ratings of 1 to 3)		0.48	0.26	3.27	0.071	1.61	0.96 to 2.70
Neutral (rating of 4)		−0.32	0.27	1.45	0.229	0.73	0.43 to 1.22
Agree (ratings 5 to 7)		REF	-	-	-	-	-
Opinion of Non-Consented Reporting	0.02						
Disagree (ratings of 1 to 3)		0.35	0.19	3.65	0.056	1.43	0.99 to 2.05
Neutral (rating of 4)		0.25	0.22	1.31	0.253	1.29	0.84 to 1.98
Agree (ratings 5 to 7)		REF	-	-	-	-	-
Opinion of Consented Reporting	0.01						
Disagree (ratings of 1 to 3)		−0.24	0.32	0.56	0.456	0.79	0.43 to 1.47
Neutral (rating of 4)		0.31	0.33	0.89	0.347	1.36	0.72 to 2.59
Agree (ratings 5 to 7)		REF	-	-	-	-	
I disclosed being sexually harassed by a faculty member							
Knowledge of Reporting Requirement	0.01						
Disagree (ratings of 1 to 3)		−0.12	0.37	0.10	0.748	0.89	0.43 to 1.84
Neutral (rating of 4)		−0.08	0.34	0.05	0.820	0.93	0.47 to 1.82
Agree (ratings 5 to 7)		REF	-	-	-	-	-
Opinion of Non-Consented Reporting	0.01						
Disagree (ratings of 1 to 3)		−0.06	0.24	0.07	0.788	0.94	0.59 to 1.50
Neutral (rating of 4)		−0.02	0.27	0.01	0.945	0.98	0.58 to 1.66
Agree (ratings 5 to 7)		REF	-	-	-	-	-
Opinion of Consented Reporting	0.01						
Disagree (ratings of 1 to 3)		−0.02	0.34	0.00	0.961	0.98	0.51 to 1.91
Neutral (rating of 4)		−0.03	0.43	0.01	0.938	0.97	0.42 to 2.22
Agree (ratings 5 to 7)		REF	-	-	-	-	-
I disclosed being raped by another student							
Knowledge of Reporting Requirement	0.01						
Disagree (ratings of 1 to 3)		0.39	0.29	1.87	0.172	1.48	0.84 to 2.59
Neutral (rating of 4)		0.12	0.30	0.15	0.700	1.12	0.62 to 2.02
Agree (ratings 5 to 7)		REF	-	-	-	-	-
Opinion of Non-Consented Reporting	0.00						
Disagree (ratings of 1 to 3)		0.24	0.21	1.37	0.242	1.28	0.85 to 1.92
Neutral (rating of 4)		−0.05	0.26	0.04	0.849	0.95	0.57 to 1.59
Agree (ratings 5 to 7)		REF	-	-	-	-	-
Opinion of Consented Reporting	0.02						
Disagree (ratings of 1 to 3)		0.24	0.34	0.50	0.480	1.27	0.65 to 2.49
Neutral (rating of 4)		−0.20	0.38	0.28	0.598	0.82	0.39 to 1.72
Agree (ratings 5 to 7)		REF	-	-	-	-	-
I disclosed being raped by a faculty member							
Knowledge of Reporting Requirement	0.00						
Disagree (ratings of 1 to 3)		−0.02	0.35	0.00	0.965	0.99	0.50 to 1.96
Neutral (rating of 4)		−0.10	0.38	0.06	0.800	0.91	0.43 to 1.93
Agree (ratings 5 to 7)		REF	-	-	-	-	-
Opinion of Non-Consented Reporting	0.00						
Disagree (ratings of 1 to 3)		−0.11	0.26	0.18	0.672	0.90	0.54 to 1.50
Neutral (rating of 4)		−0.39	0.28	1.89	0.169	0.68	0.39 to 1.18
Agree (ratings 5 to 7)		REF	-	-	-	-	-
Opinion of Consented Reporting	0.01						
Disagree (ratings of 1 to 3)		−0.16	0.36	0.21	0.646	0.85	0.42 to 1.70
Neutral (rating of 4)		−0.32	0.49	0.43	0.513	0.72	0.28 to 1.91
Agree (ratings 5 to 7)		REF	-	-	-	-	-
If you were sexually assaulted by another student, how likely would you be to tell a faculty member about the incident?	0.02						
Would not disclose (ratings of 1 or 2)		0.74	0.20	13.06	< 0.001	2.09	1.40 to 3.12
Unsure (rating of 3)		0.29	0.18	2.74	0.098	1.34	0.95 to 1.88
Would disclose (ratings of 4 or 5)		REF	-	-	-	-	-
If you knew that an acquaintance of yours had sexually assaulted another student, how likely would you be to tell a faculty member about the incident?	0.02						
Would not disclose (ratings of 1 or 2)		0.41	0.22	3.41	0.065	1.51	0.98 to 2.34
Unsure (rating of 3)		0.35	0.17	4.34	0.037	1.42	1.02 to 1.97
Would disclose (ratings of 4 or 5)		REF	-	-	-	-	-
Is the likelihood of you telling a trusted faculty member if you were sexually assaulted affect by Title IX reporting requirements?	0.02						
Decreased likelihood (ratings of 1 or 2)		0.75	0.23	10.61	0.001	2.12	1.35 to 3.34
Unchanged (rating 3)		0.18	0.18	1.02	0.312	1.20	0.84 to 1.71
Increase likelihood (ratings of 4 or 5)		REF	-	-	-	-	-

Note: Sex was included as a covariate. *SE* = standard error; *OR* = odds ratio; *CI* = confidence interval.
